# Editorial: Cellular dysfunction and death in diabetes (etio)pathology: Novel insights into molecular mechanisms and therapeutic targeting

**DOI:** 10.3389/fendo.2022.1121518

**Published:** 2023-01-10

**Authors:** M. Markelic, S. Batirel, A. Stancic

**Affiliations:** ^1^ Faculty of Biology, University of Belgrade, Belgrade, Serbia; ^2^ Faculty of Medicine, Marmara University, Istanbul, Türkiye; ^3^ Institute for Biological Research “Sinisa Stankovic” - National Institute of the Republic of Serbia, University of Belgrade, Belgrade, Serbia

**Keywords:** diabetes, diabetic complications, ferroptosis, islet amyloid polypeptide, beta cell death

The increase in the incidence of diabetes requires major research and clinical efforts to reduce the prevalence and progression of this disease. Most of the therapeutic approaches used in clinical practice have a reparative effect to correct already developed pathologies or to slow their progression. A comprehensive understanding of the underlying mechanisms of the etiology and pathogenesis of diabetes remains necessary to intervene efficiently and rapidly. In this context, increased production of reactive oxygen species (ROS) has been identified as a key event. Indeed, oxidative stress is the link between the major pathological features of diabetes (hyperglycemia, hyperlipidemia-induced metabolic stress, and cytokine-induced inflammatory stress) and the consequent cell damage and death. The decrease in the functional population of β-cells is the crucial process for the initiation of type 1 diabetes (T1D) and for the progression of type 2 diabetes (T2D). Cell death also underlies the progression of diabetic complications, culminating in damage to many tissues and organs such as the liver, heart, retina, kidneys, and nervous system. Thus, targeting redox-sensitive signaling pathways and cell death represents a promising approach for reducing the onset and improving the course of diabetes. To date, the involvement of different types of regulated cell death (apoptosis, necrosis, autophagy, and ferroptosis) in diabetes and diabetes-related diseases has been demonstrated. There is growing evidence that correcting ROS production and ROS-induced cell damage/death may be an effective approach for the prevention and treatment of diabetes and diabetic complications. However, success in clinical practice is yet to come.

The articles in this Research Topic covered a variety of aspects of diabetes investigations: i) underlying molecular mechanisms of diabetic complication; ii) cell death involved in the demise of β-cells and other cells affected in diabetes; iii) potential approaches to prevent and treat diabetes-related cell injury/death; iv) current challenges in the treatment of diabetes and diabetic complications.

The review paper by Yang and Liu aimed to present epidemiological and experimental data on diabetic nephropathy and retinopathy (DN and DR, respectively). These commonly observed diabetic complications were discussed in terms of microvascular endothelial cell dysfunction, which is a common consequence of diabetes-induced hyperglycemia and downstream signaling dysfunction. Initially, the authors focused on the classic regulators of endothelial dysfunction in DN and DR, such as disturbances in signaling of cell adhesion molecules, vascular endothelial growth factor, Notch, and ROS. Importantly, the authors provided relevant literature evidence that highlighted new research priorities in this area, including exosomes, circular RNA, and interaction with neighboring cells. Finally, the authors described the current therapy for DN and DR and emphasized the newly opened avenues for its improvement based on the mechanisms discussed in the review paper.

The review by Yang and Yang focused on the involvement of ferroptosis in the pathogenesis of diabetes and diabetes-related complications. After a brief overview of ferroptosis, in which the authors describe the morphological and biochemical features of this type of cell death, they provide evidence for its contribution to disorders of glucose and lipid metabolism. Another focus is the role of ferroptosis in various diabetes-related metabolic complications such as DN, DR, neuropathy, osteoporosis, and cardiomyopathy. The possibility of using synthetic and natural ferroptosis inhibitors in the treatment of diabetes was also discussed. In conclusion, it was emphasized that further studies are needed to explore the underlying pathophysiological mechanisms and to develop appropriate ferroptosis-targeted antidiabetic therapy.

In the review by  Dinic et al. literature data supporting oxidative stress as an important target in the treatment of diabetes, particularly with regard to β-cell survival, were presented. As a starting point, the authors described redox-related mechanisms involved in β-cell death and dysfunction in T1D (resulting from inflammation) and T2D (resulting from insulin resistance and glucotoxicity). Oxidative stress-induced de/transdifferentiation of β-cells and the possibility of using antioxidants to preserve β-cell identity and function in the diabetic state were analyzed. Also, the relationship between oxidative stress and epigenetic changes in β-cells and the success of using natural antioxidants to combat them was discussed. Special attention was paid to the application of state-of-the-art CRISPR/Cas9 technology based on targeted epigenome editing with the aim of altering the differentiation state of various cell types and turning them into insulin producers. In the end, the authors propose targeted epigenetic therapies as a promising approach for the treatment of various diseases, including diabetes.

In their original work, Raimundo et al. focus on the antidiabetic strategy based on the prevention/treatment of the aggregates of a pancreatic hormone amylin, i.e., islet amyloid polypeptide (IAPP) within β-cells. As authors emphasized, IAPP, especially in its immature form, has amyloidogenic properties, and its deposition is recognized as an important mediator and pathohistological marker of β-cell dysfunction and death in T2D. The focus of this study are metabolites of (poly)phenols as potential inhibitors of IAPP aggregation. Therefore, Raimundo et al. developed a screening strategy to test a collection of polyphenol metabolites. Based on *in silico* (molecular docking), cell-free, and yeast model studies of IAPP aggregation, the authors identified Urolithin B, a small polyphenol metabolite (a product of colon metabolism of dietary ellagic acid) as a potent inhibitor of IAPP aggregation and a stimulator of its clearance *via* the autophagy. Overall, the results of this study shed new light on this (poly)phenol metabolite, as a potential antidiabetic nutraceutical.

In addition to drug treatment of diabetes, efforts to develop regenerative approaches have been ongoing in recent years, as reported by Wang et al. To provide a sufficient source of β-cells, treatment with pluripotent stem cells (PSCs), especially induced PSCs (iPSCs), has received special attention because the use of human embryonic PSCs (hESCs) is restricted due to ethical concerns. This review summarizes pioneering and current protocols for differentiation of PSCs into β-cells, generation of islet organoids, and current strategies for their successful transplantation. Ongoing clinical trials based on PSC therapy, speak to the tremendous progress made in the field of regenerative antidiabetic approaches. However, as discussed by Wang et al. important questions remain to be addressed regarding maturation and phenotype of β-cells, vascularization of grafts, immune rejections and other health concerns.

Overall, the articles published in this Research Topic highlight the broad scope of research being conducted to advance our understanding of the cellular and molecular processes involved in diabetes (etio)pathology and advance the discovery of novel antidiabetic therapies.

## Author contributions

All authors listed have made a substantial, direct, and intellectual contribution to the work and approved it for publication.

